# Asexual Development and Virulence Are Not Affected by Knockout of Five Sexual-Stage Candidate Genes in *Toxoplasma gondii*

**DOI:** 10.3390/ani16172704

**Published:** 2026-08-31

**Authors:** Xing Tian, Chen-Ran Tian, Xin-Sheng Lu, Wen-Bo Hao, Zhi Zheng, Jun-Jun He, Xing-Quan Zhu, Xiao-Nan Zheng

**Affiliations:** 1 Shanxi Key Laboratory of Animal Disease Research, Prevention and Control, College of Veterinary Medicine, Shanxi Agricultural University, Jinzhong 030801, China; txing2001@163.com (X.T.); chenrantian2019@163.com (C.-R.T.); luxinsheng2024@126.com (X.-S.L.); 16696739873@163.com (W.-B.H.); 15863201790@163.com (Z.Z.); 2 The Yunnan Key Laboratory of Veterinary Etiological Biology, College of Veterinary Medicine, Yunnan Agricultural University, Kunming 650201, China; hejunjun617@163.com

**Keywords:** *Toxoplasma gondii*, CCCH-type zinc finger protein, subtilisin-like protease, CRISPR-Cas9, phenotype

## Abstract

*Toxoplasma gondii* is a widespread intracellular parasite posing serious risks to immunocompromised individuals and pregnant women, and causing significant economic losses to the livestock industry. Current clinical drugs mainly target acute-stage tachyzoites but are ineffective against chronic cysts, necessitating new interventions. The parasite’s sexual stage in the feline intestine remains poorly understood but is critical for transmission. Here, based on our unpublished weighted gene co-expression network analysis of single-cell transcriptomic data of cat intestine after infection with *T. gondii*, we selected five previously uncharacterized genes, including three CCCH-type zinc finger proteins (*TGME49_257130*, *TGME49_500318*, *TGME49_313740*) and two subtilisin-like proteases (*TGME49_248760*/*sub7*, *TGME49_235950*/*sub8*). Using the CRISPR-Cas9 system, individual knockout strains were generated from the type II Pru strain. And, we performed phenotypic assessments at this stage as an essential validation to confirm neutrality before feline infection experiments. Phenotypic analyses revealed that none of the knockout strains showed significant differences in vitro growth, replication, egress, in vivo virulence, or cyst formation from the wild-type strain, corroborating the transcriptional prediction. Publicly available ToxoDB transcriptomic data confirmed that four of these genes were highly expressed at the oocyst or feline enteroepithelial stages, but barely expressed in tachyzoites and bradyzoites, consistent with our phenotypic observations. Collectively, these findings indicate that these genes are dispensable for asexual development, while their expression pattern suggests potential involvement in sexual reproduction, a hypothesis that warrants direct experimental validation. This study provides validated knockout strains and candidate genes, establishing an experimental foundation for future functional studies on *Toxoplasma* sexual development and transmission-blocking strategies.

## Simple Summary

*Toxoplasma gondii* is a common parasite that infects humans and nearly all birds and mammals, posing a serious threat to immunocompromised individuals and pregnant women, and causing significant economic losses to the livestock industry. Its sexual reproduction occurs exclusively in cat intestines, a poorly understood stage critical for transmission. Based on our single-cell transcriptomic data from infected cat intestines, we selected five previously uncharacterized genes that are highly expressed in sexual stages, including three zinc finger protein genes and two subtilisin-like protease genes. Using CRISPR-Cas9 gene-editing technology, we successfully constructed knockout mutants and systematically evaluated their tachyzoite phenotypes to confirm phenotypic neutrality before future feline infection experiments. The results show that knockout of these five genes did not significantly affect in vitro parasite growth, virulence in mice, or brain cyst formation, corroborating the transcriptional predictions. Analysis of publicly available ToxoDB transcriptomic data confirmed the expression patterns of four of these genes observed in our previous analysis. These findings indicate that the five genes are dispensable for the asexual reproduction and virulence of *T. gondii* and suggest that these genes may be involved in sexual reproduction within the cat intestine, a possibility that requires direct experimental validation. This study provides well-characterized knockout strains and candidate genes, establishing a foundation for investigating sexual-stage biology and developing transmission-blocking strategies against *T. gondii* infection.

## 1. Introduction

*Toxoplasma gondii* is an obligate intracellular apicomplexan parasite with a worldwide distribution, capable of infecting nearly all warm-blooded animals, including humans [1,2]. Seroprevalence surveys indicate that approximately one-third of the global human population has been infected with this parasite [3]. *T. gondii* exhibits a complex life cycle, involving asexual reproduction in intermediate hosts (including humans and other warm-blooded animals) and sexual reproduction within the intestinal epithelial cells of definitive hosts (felids) [4]. In intermediate hosts, rapidly proliferating tachyzoites cause acute infection. Under host immune pressure, tachyzoites can convert into bradyzoites, forming tissue cysts that are most frequently found in the brain and skeletal muscle, establishing lifelong chronic infection [5,6]. For individuals with weakened immune systems, such as organ transplant recipients and AIDS patients, both initial *Toxoplasma* infection and reactivation of latent toxoplasmosis may cause life-threatening toxoplasmic encephalitis as well as other critical complications [7,8]. Moreover, primary infection in pregnant women can be vertically transmitted to the fetus, resulting in miscarriage, stillbirth, or congenital toxoplasmosis, which causes neurological and ocular abnormalities in newborns [9]. Currently, most clinical anti-*Toxoplasma* drugs primarily target acute stage tachyzoites, exhibit limited efficacy against chronic cysts and cause significant adverse effects, while effective vaccines remain unavailable [2,4]. Elucidating the molecular mechanisms governing parasite pathogenesis and development, particularly the largely unresolved events of sexual reproduction in the definitive host, is of great scientific value and will provide a crucial basis to develop novel strategies for intervention [10,11,12].

The zinc finger proteins constitute one of the most widespread protein families in eukaryotes. Their characteristic domains form stable three-dimensional conformations through coordination of zinc ions with conserved cysteine and histidine residues, mediating interactions with DNA, RNA, or other proteins [13,14], and are involved in a wide range of cellular events, such as DNA damage repair, RNA turnover, transcriptional modulation and cellular signal transduction [15]. Based on the combination of zinc-coordinating residues, zinc finger proteins are classified into C2H2-type, CCCH-type, RING-type, PHD-type, and others. Among them, CCCH-type zinc finger proteins are important RNA-binding proteins that regulate target mRNA stability and translation in apicomplexan parasites and immune cells [13,16]. Several zinc finger proteins identified in *Toxoplasma* play critical roles in cell division, stage differentiation, and RNA metabolism. TgZFP1 was the first nucleolus-localized CCCH-type zinc finger protein characterized in *Toxoplasma*, and the disruption of the upstream region of this gene significantly reduces the efficiency of the parasite’s stage transition [17]. TgZFP2 contains a conserved zinc ribbon domain, and conditional knockout of TgZFP2 leads to cell cycle arrest and failure to form complete daughter parasites [18]. TgZNF2 is a nuclear protein, and inducible knockout of this gene causes developmental arrest at the G1 phase [19]. As a class of crucial post transcriptional regulators, the functional roles of other CCCH-type zinc finger proteins in *Toxoplasma* remain largely unexplored.

In addition to zinc finger proteins, another family of proteases—subtilisin-like serine proteases—also plays crucial roles in parasite biology. Subtilisin-like serine proteases (SUBs) are a family of serine proteases widely distributed across biological kingdoms. Their catalytic triad is arranged in the amino acid sequence as aspartate-histidine-serine (Asp-His-Ser), a structural feature that distinguishes them as a unique protease family [20,21]. In apicomplexan parasites, they participate in proteolytic processing and maturation of proteins [20,21]. In *Toxoplasma*, several SUB family members have been functionally characterized. TgSUB1 localizes to micronemes and processes microneme proteins (e.g., M2AP, MIC2, MIC4), playing a key role during host cell invasion [22]. TgSUB2 localizes to rhoptries and is autocatalytically processed during its transport along the secretory pathway [23,24]. TgSUB3 deletion severely impairs parasite proliferation [25]. However, the biological functions of TgSUB7 (*TGME49_248760*) and TgSUB8 (*TGME49_235950*), two additional members of the SUB family, have not yet been reported.

In this study, we selected five genes of interest (GOIs), including three CCCH-type zinc finger proteins (*TGME49_257130*, *TGME49_500318*, *TGME49_313740*) and two subtilisin-like proteases (*TGME49_248760*/*sub7, TGME49_235950*/*sub8*), which were identified from our unpublished weighted gene co-expression network analysis (WGCNA) of single-cell transcriptomic data of cat intestine following *T. gondii* infection. These genes were prioritized because they exhibited significantly higher expression in sexual stages and possessed unknown phenotypes. Since CRISPR-Cas9 transfection and subsequent drug selection are efficiently performed in tachyzoite stage, genetic manipulation in tachyzoites serves as a technical prerequisite for generating knockout mutants for downstream feline infection experiments to examine their sexual-stage functions. Accordingly, tachyzoite phenotyping in this study primarily serves as a validation step, specifically confirming successful gene disruption and establishing baseline phenotypes. Using the CRISPR-Cas9 gene editing system, we generated individual knockout strains for each gene in the type II Pru background and systematically assessed their phenotypes, including plaque formation, intracellular replication, egress, virulence evaluation in a mouse model, and brain cyst enumeration during chronic infection.

## 2. Materials and Methods

### 2.1. Mice and Parasites

The human foreskin fibroblast (HFF) cell line (HFF-1) used in the present study was purchased from the American Type Culture Collection (ATCC; Cat. No. SCRC-1041™; Manassas, VA, USA), and preserved in the Shanxi Key Laboratory of Animal Disease Research, Prevention and Control, College of Veterinary Medicine, Shanxi Agricultural University. HFF cells were cultured in DMEM (Gibco, Suzhou, China) containing 10% fetal bovine serum (FBS; Gibco, Thornton, NSW, Australia), 100 U/mL penicillin, and 100 μg/mL streptomycin at 37 °C under 5% CO_2_-humidified conditions. The parasite strain used in this study was *T. gondii* type II PruΔ*ku80*Δ*hxgprt* tachyzoites (referred to as Pru). Tachyzoites were cultured in HFF monolayers using DMEM containing 2% FBS under the same conditions as those for HFF cells. To collect parasites, intracellular parasites were released by repeated pipetting with a 27-gauge needle. The suspension was filtered through a 5 μm polycarbonate membrane to remove cellular debris, yielding purified tachyzoites for subsequent experiments [10,26].

### 2.2. Construction of CRISPR-Cas9 Knockout Vectors

In this study, we used CRISPR-Cas9-mediated homologous recombination to knock out the five GOIs. For each target gene, a specific single guide RNA (sgRNA) was designed against the coding region. Using the pSAG1::Cas9-U6::sgUPRT plasmid as a template, the original guide RNA targeting the UPRT gene was replaced with the specific sgRNA for each target gene to construct the corresponding CRISPR knockout plasmid [26].

For donor plasmid construction, the 5′ upstream (~1200 bp) and 3′ downstream (~1200 bp) homology arms (5′UTR and 3′UTR) of each target gene were amplified from *T. gondii* type II Pru strain genomic DNA. The DHFR expression sequence was amplified from the pUPRT-DHFR-D plasmid. Using the Clone Express II One Step Cloning Kit (Vazyme, Nanjing, China), the three fragments were ligated with the pUC19 backbone to construct the donor plasmid pUC19-5′UTR-DHFR-3′UTR for each target gene. The sequence-verified donor plasmid served as a template to amplify the 5′UTR-DHFR-3′UTR homologous recombination template fragment with specific primers, which was then purified and stored at −20 °C for later use. Details of the primers used for constructing the knockout plasmids and homologous templates are provided in Table S1.

### 2.3. Parasite Transfection and Screening of Knockout Strains

Freshly egressed Pru tachyzoites were mixed with CRISPR knockout plasmid (~35 μg) and the amplified donor fragment (~20 μg), electroporated and subsequently inoculated into HFF cells [26]. At 24 h post transfection, pyrimethamine was added to a final concentration of 3 μM for drug selection. Once resistant parasite populations emerged, parasites were collected and serially diluted in 96-well plates. After 7–10 days, genomic DNA was extracted from each monoclonal parasite clone. Three pairs of primers were designed for PCR identification: PCR1 (5′ homology arm outer forward primer and DHFR internal reverse primer) and PCR3 (DHFR internal forward primer and 3′ homology arm outer reverse primer) were used to detect homologous recombination; PCR2 (internal primers within the target gene coding region) was used to verify gene deletion. Correctly identified knockout strains were expanded and used for subsequent experiments. The detailed information of the identification primers is listed in Table S1.

### 2.4. Plaque Assay

HFF cells were seeded in 12-well plates and cultured until confluent. 200 tachyzoites (wild-type Pru or each knockout strain) were added per well and cultured statically at 37 °C with 5% CO_2_ for 10 days without medium replacement. Following incubation, the medium was removed, and the wells were gently rinsed three times with PBS. Cells were fixed with 4% paraformaldehyde (Solarbio, Beijing, China) for 30 min and subsequently stained with 0.5% crystal violet (Solarbio, Beijing, China) for 30 min. Excess dye was removed with PBS, and plates were air-dried at room temperature. Plaques were photographed using a digital camera (Nikon Z6 II, Nikon Corporation, Tokyo, Japan) equipped with a macro lens. The acquired images were subsequently processed and captured using Adobe Photoshop software (Version 26.0.0) (Adobe Inc., San Jose, CA, USA). The number and area of plaques per well were quantified using ImageJ version 1.54p (National Institutes of Health, Bethesda, MD, USA). For each strain, the area of individual plaques was quantified, and the mean plaque size was normalized to that of the wild-type Pru strain, which was set as 100%. The normalized data were used for comparative analysis. Each experiment included triplicate wells and was repeated three times independently [10].

### 2.5. Intracellular Replication and Egress Assays

HFF cells were seeded in small dishes until confluent and inoculated with 5 × 10^5^ tachyzoites per dish. After 2 h of incubation at 37 °C with 5% CO_2_ to allow invasion, the dishes were washed three times with pre-warmed PBS to remove non-invading extracellular parasites, and fresh medium was added. After 32 h of culture, the medium was removed, and cells were fixed with 4% paraformaldehyde for 30 min, permeabilized with 0.1% Triton X-100 for 10 min, and blocked with 3% BSA (VWR Chemicals, Solon, OH, USA) for 1 h. Mouse anti-SAG1 antibody (1:500 dilution, Thermo Fisher Scientific, Rockford, IL, USA, Cat# MA5-18268) was added and incubated overnight at 4 °C. After three washes with PBS, Alexa Fluor 488-conjugated goat anti-mouse secondary antibody (1:500 dilution, Thermo Fisher Scientific, Rockford, IL, USA, Cat# A32723) was added and incubated for 1 h at 37 °C. No fewer than 100 parasitophorous vacuoles (PVs) were randomly examined under fluorescence microscopy, and the quantity of tachyzoites (2, 4, 8, 16, 32) within each PV was documented. For egress assays, parasites were cultured for 44 h until most vacuoles contained 16 or 32 parasites. The medium was then replaced with DMEM containing 3 μM calcium ionophore A23187 (Sigma, Burlington, MA, USA, Cat# C7522), and dishes were incubated at 37 °C for 2.5 min to induce egress. Immediately after, the medium was removed, and cells were fixed with 4% paraformaldehyde for 30 min, followed by three PBS washes. At least 100 vacuoles were randomly counted to determine the number of egressed and non-egressed vacuoles, and egress rates were calculated. Each experiment was performed in triplicate wells and repeated three times independently [10].

### 2.6. Virulence in Mice and Brain Cyst Enumeration

To evaluate the virulence of each knockout strain, six- to eight-week-old female specific-pathogen-free (SPF) Kunming mice purchased from Beijing Sibeifu Biotechnology Co., Ltd. (Beijing, China) were randomly allocated into two groups (*n* = 6 per group). Freshly purified tachyzoites of each knockout strain and the wild-type Pru strain were diluted with PBS to the desired concentrations. Each mouse received an intraperitoneal injection of 200 μL of suspension containing either 5 × 10^5^ or 5 × 10^3^ tachyzoites. After infection, mice were monitored daily for clinical symptoms and mortality for 30 consecutive days. Survival times were recorded, and survival curves were plotted.

Enumeration of brain cysts is a common approach for quantifying chronic infection in murine models [4]. At 30 days post-infection, surviving mice were euthanized. Cyst counts were performed only on mice that survived to the 30-day endpoint. Whole brains were aseptically removed and homogenized thoroughly using a mortar and pestle. PBS was added to prepare 1.5 mL of brain homogenate suspension. A 10 μL aliquot of the homogenate was placed on a glass slide and covered with a coverslip and eight replicate slides were prepared per sample. Intact cysts were counted under a light microscope. The average number of cysts from the eight slides was used to calculate the total number of cysts per whole brain based on the total homogenate volume [26].

All animal experiments described in this study were conducted under the approval of the Institutional Animal Care and Use Committee of Shanxi Agricultural University (Approval No. SXAU-EAW-2020ME8.OJ.005018326), and all procedures were performed within the validity period of this approval. Mice were housed under specific-pathogen-free conditions with a 12 h light/dark cycle at 22 ± 2 °C and 50–60% relative humidity, with free access to food and water. Prior to the experiments, all mice were acclimatized to the housing environment for one week. Throughout the study, all efforts were made to minimize animal suffering and ensure animal welfare, and all procedures were performed in accordance with institutional and national guidelines for the care and use of laboratory animals.

### 2.7. Bioinformatic Analysis of Four Novel Toxoplasma Genes

To investigate the detailed information of the four GOIs, we retrieved and re-analyzed publicly available data from the ToxoDB database (ToxoDB release 68; https://toxodb.org; accessed on 1 March 2026). This study analyzed the genomic characteristics of four GOIs: molecular weight, phenotype values, exon count, transmembrane domains (TMHMM prediction), and predicted signal peptides. Additionally, transcriptomic data of four genes were collected under different genotypes, cell cycle stages, bradyzoite differentiation times, and developmental phases. No transcriptomic data were available for *TGME49_500318* in the ToxoDB database at the time of analysis.

### 2.8. Statistical Analysis

All in vitro experiments were performed with three independent biological replicates. For the in vivo virulence and cyst formation assays, each experimental group contained six mice (*n* = 6) per dose, and the data represent a single cohort experiment conducted in accordance with standard animal challenge protocols. Statistical analyses were conducted using GraphPad Prism 10.1.2 (GraphPad Software, Boston, MA, USA). Data normality was assessed using the Shapiro–Wilk test, and homogeneity of variances was evaluated using the Brown–Forsythe test. For comparisons involving normally distributed data with equal variances, one-way ANOVA followed by Dunnett’s multiple comparisons test was used. For comparisons involving normally distributed data with unequal variances, the Welch ANOVA followed by Dunnett T3 multiple comparisons test was used. For data that did not follow a normal distribution, the Kruskal–Wallis test followed by Dunn’s multiple comparisons test was used. For survival curve comparisons, the log-rank (Mantel–Cox) test was used. Values are shown as mean ± standard deviation (SD). Differences were considered statistically significant at *p* < 0.05. Significance levels are indicated as *p* ≥ 0.05 (ns), *p* < 0.05 (*), *p* < 0.01 (**).

## 3. Results

### 3.1. Successful Generation of Knockout Strains

**Figure 1:**
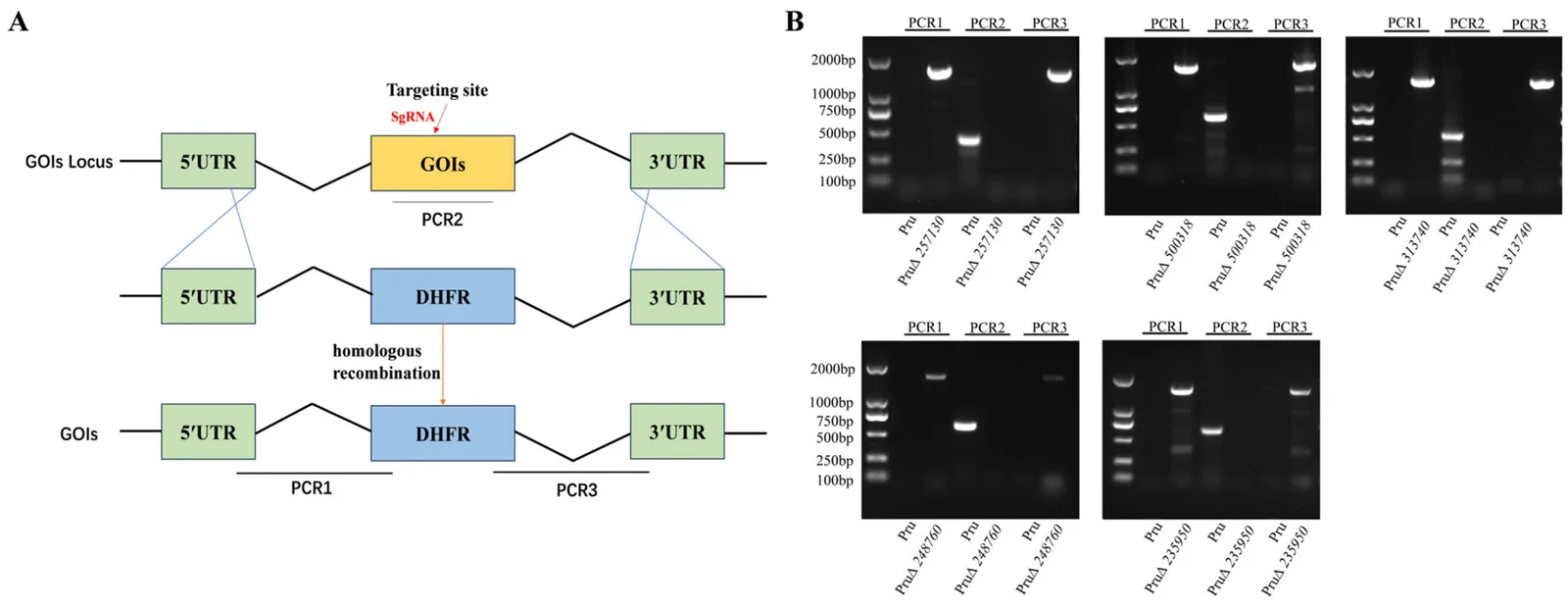
Construction of five PruΔ*GOI* strains. (**A**) Schematic representation illustrating the deletion of genes of interest (GOIs) using the CRISPR-Cas9 system. (**B**) Validation of the five PruΔ*GOI* strains by diagnostic PCR tests. Each panel represents the identification image of PruΔ*257130*, PruΔ*500318*, PruΔ*313740*, PruΔ*248760*, and PruΔ*235950*, respectively. PCR1 and PCR3 were respectively designed to validate homologous fragment integration into the 5′ and 3′ flanking regions of the target genes. PCR2 was employed to confirm that the GOIs were successfully replaced by the DHFR cassette.

Five previously uncharacterized genes were selected in this study, including three zinc finger protein genes and two subtilisin-like protease genes (Table 1). We used the CRISPR-Cas9 system to replace the coding region of each GOI with the corresponding homologous fragment (5′UTR-DHFR-3′UTR) (Figure 1A) and successfully knocked out each individual gene in the type II Pru strain. PCR identification was performed on the knockout strains. PCR1 and PCR3 were employed to confirm the insertion site of the homologous fragments, and the results showed that a fragment of approximately 1500–1800 bp was amplified in each GOI knockout strain, indicating that the homologous fragments had been successfully integrated into the target gene locus. PCR2 was used to amplify the coding sequence and the expected target fragment (approximately 400–600 bp) was detected in the wild-type strain, while no amplification product was observed in the knockout strains, further confirming the knockout of the target gene. The PCR results verified that the five Pru knockout strains (PruΔ*257130*, PruΔ*500318*, PruΔ*313740*, PruΔ*sub7*, and PruΔ*sub8*) were successfully constructed (Figure 1B).

### 3.2. Knockout Strains Show No Obvious Growth Defects In Vitro

**Figure 2:**
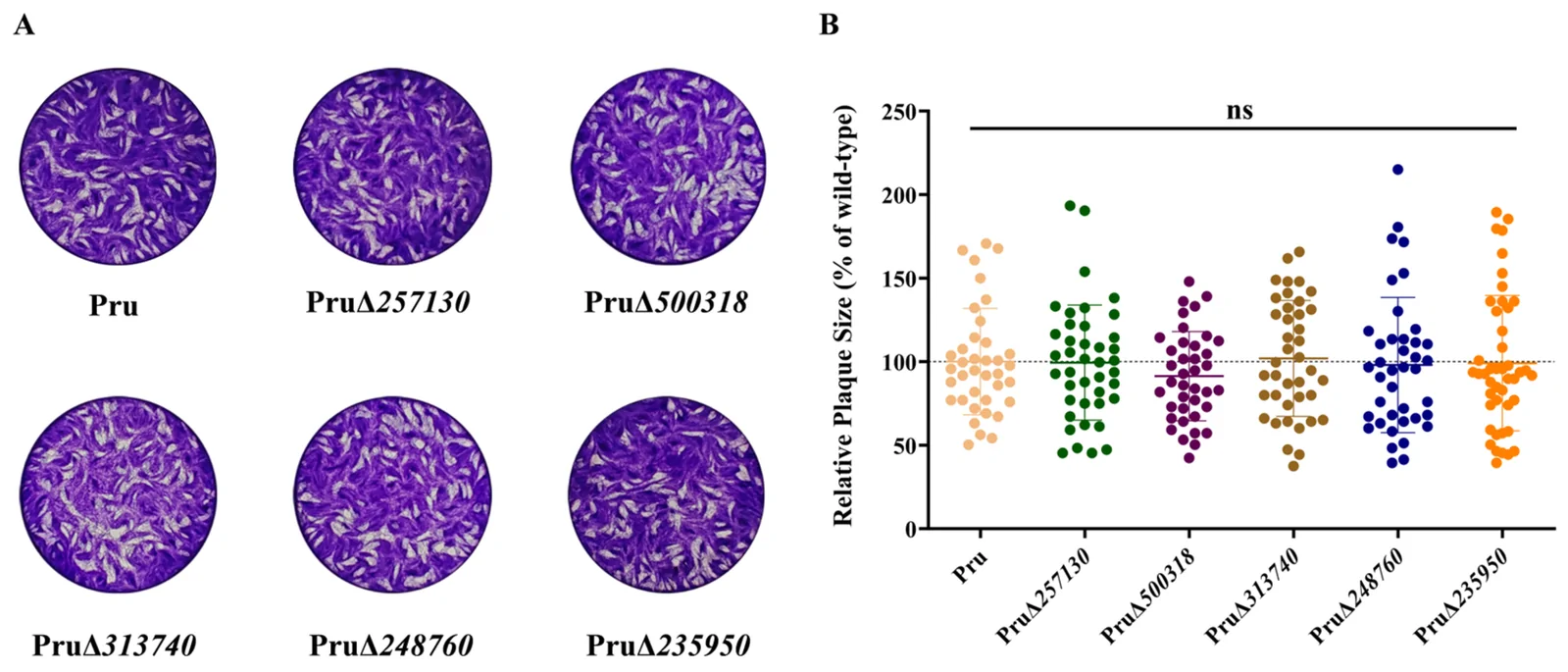
Evaluation of the lytic cycle in five PruΔ*GOI* and wild-type *T. gondii* strains. (**A**) Representative plaque assay images of the indicated strains after 10 days of growth in HFFs, from three independent experiments. (**B**) Relative size analysis of the plaque assays. The mean plaque size was normalized to the wild-type Pru strain (set as 100%). Statistical analysis was performed using the Kruskal–Wallis test with Dunn’s multiple comparisons test due to non-normal distribution, with the wild-type Pru strain as the control. ns, not significant (*p* ≥ 0.05).

To evaluate the impact of single deleting the five genes on tachyzoite growth in vitro, we performed plaque formation, intracellular replication, and egress assays. Plaque assay data demonstrated that the relative plaque sizes formed by the five knockout strains showed no significant difference from the wild-type Pru strain after 10 days of culture (Figure 2A,B).

**Figure 3:**
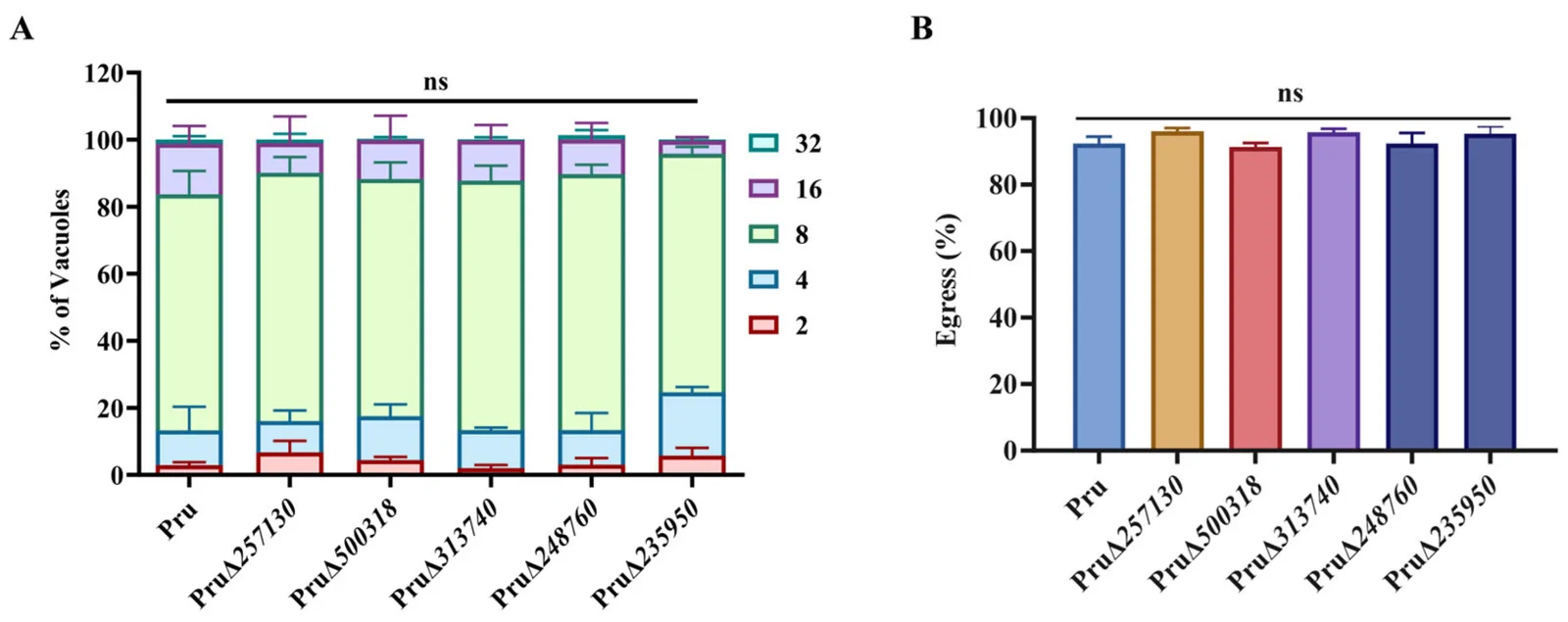
In vitro assays for intracellular replication and egress efficiency of PruΔ*GOI* strains. (**A**) Intracellular replication of the indicated strains at 32 h post-infection. The distribution of vacuoles containing 2, 4, 8, 16, or 32 tachyzoites is shown. Data are presented as mean ± SD from three independent experiments, each performed in triplicate. Statistical analysis was performed using one-way ANOVA with Dunnett’s multiple comparisons test due to normal distribution. No significant differences were observed between any knockout strain and the Pru strain at any vacuole category (*p* ≥ 0.05). (**B**) Egress efficiency of the indicated strains following calcium ionophore A23187 induction. Data are presented as mean ± SD from three independent experiments, each performed in triplicate. Statistical analysis was performed using the Kruskal–Wallis test with Dunn’s multiple comparisons test due to non-normal distribution, with the wild-type Pru strain as the control. ns, not significant (*p* ≥ 0.05).

Intracellular replication assays revealed that the distribution of tachyzoite numbers per vacuole (proportion of vacuoles containing 2, 4, 8, 16 or 32 parasites) in knockout strains was comparable to that of the wild-type Pru strain at 32 h post-infection, indicating no impact on replication capacity (Figure 3A). In addition, microscopic examination during immunofluorescence staining of replicating parasites revealed no morphological abnormalities in tachyzoites or PVs of any of the knockout strains. In calcium ionophore-induced egress assays, the egress rates of knockout strains were also similar to those of the wild-type strain without significant difference (Figure 3B).

### 3.3. The Knockout Strains Had No Significant Defects in Virulence and Brain Cyst Formation

**Figure 4:**
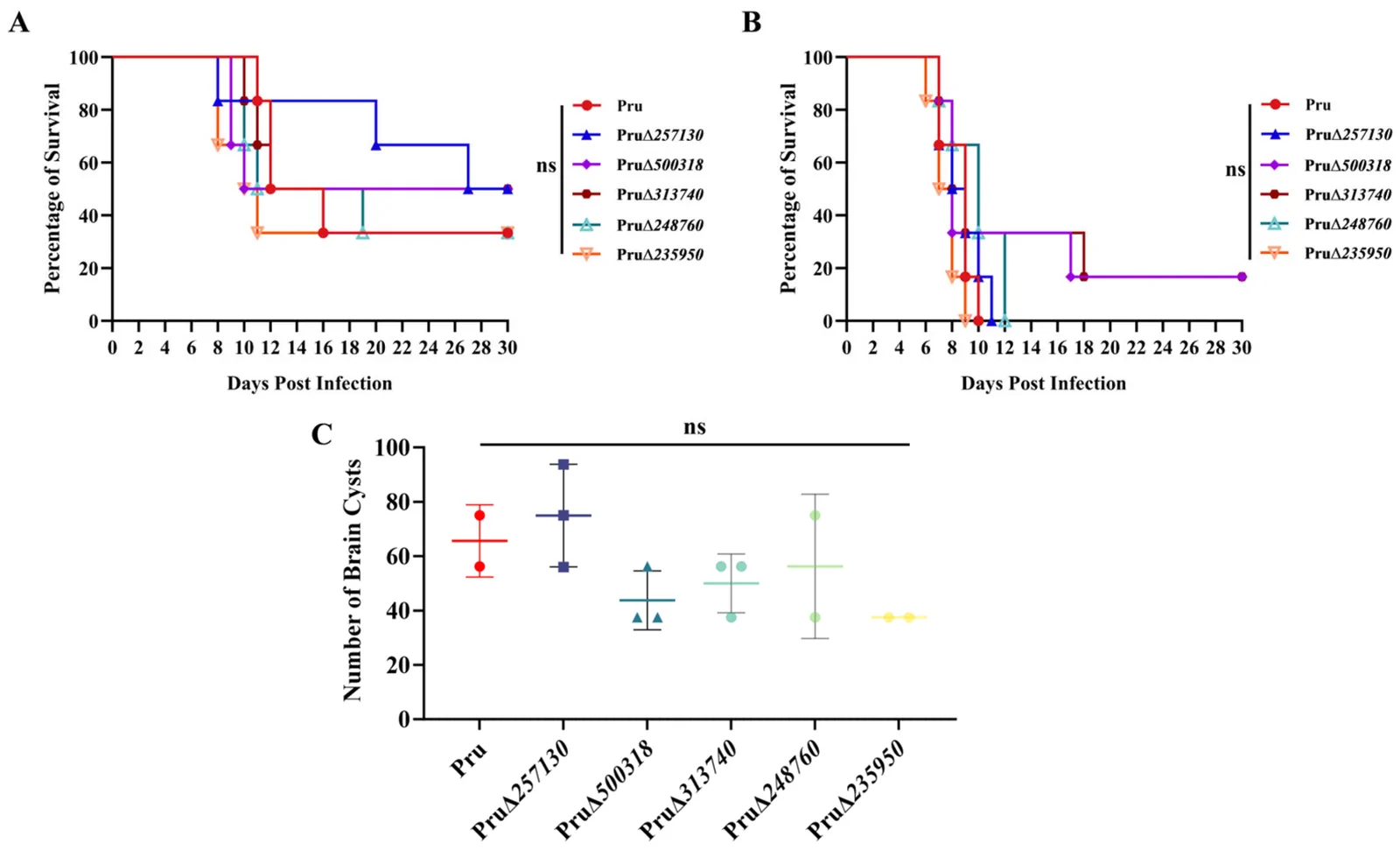
Survival and brain cyst burden in infected mice. (**A**) Survival curves of Kunming mice (*n* = 6 per strain) intraperitoneally injected with 5 × 10^3^ tachyzoites of the wild-type Pru strain or each PruΔ*GOI* strain. (**B**) Survival curves of Kunming mice (*n* = 6 per strain) intraperitoneally infected with 5 × 10^5^ tachyzoites of the wild-type Pru strain or each PruΔ*GOI* strain. For (**A**,**B**), statistical analysis was performed using the log-rank (Mantel–Cox) test. No significant differences were observed between any knockout strain and the Pru strain (*p* ≥ 0.05). (**C**) Brain cyst counts of mice that survived 30 days post-infection (from the 5 × 10^3^ tachyzoite inoculation group). Data are presented as mean ± SD. Statistical analysis was performed using the Kruskal–Wallis test with Dunn’s multiple comparisons test due to non-normal distribution, with the wild-type Pru strain as the control. ns, not significant (*p* ≥ 0.05).

To evaluate the impact of deleting the five GOIs on in vivo virulence of *T. gondii*, we performed virulence assays in mice. At both high (5 × 10^5^) and low (5 × 10^3^) inoculation doses, the survival curves of mice infected with each knockout strain showed no significant differences from those of mice infected with the wild-type Pru strain (Figure 4A,B), and the time to death was also similar to that of the wild-type Pru group.

At 30 days post-infection, surviving mice were euthanized, and brain homogenates were examined for cyst counts. In the low (5 × 10^3^) inoculation dose group, no significant differences in brain cyst numbers were observed between mice infected with any knockout strain and those infected with the wild-type Pru strain (Figure 4C). For the high-dose group (5 × 10^5^), only two mice survived to the 30-day endpoint, one in the PruΔ*500318* group and one in the PruΔ*313740* group. Brain cysts were enumerated for the surviving mouse in the PruΔ*313740* group, and no cysts were detected in eight replicate slides. The other surviving mouse (PruΔ*500318*) died on the morning of the 31st day and was not subjected to cyst enumeration. Due to the limited number of survivors in the high-dose group, statistical comparison was not performed for this dose group.

### 3.4. Four GOIs May Play Roles in Oocyst Development and Sporozoite Differentiation

We retrieved and re-analyzed publicly available transcriptomic data from the ToxoDB database for four of the five GOIs (no data were available for *TGME49_500318*) [27,28,29,30].

All four genes were stably expressed in six *T. gondii* genotypes (RH, GT1, PRU, ME49, CTG, VEG), with peak expression observed in the Pru strain (Figure S1A). In the RH strain cell cycle, the four genes exhibited periodic fluctuations, albeit with modest amplitude changes (Figure S1B). During bradyzoite differentiation, all four genes maintained stable expression levels over time (Figure S1C). Regarding developmental stages, *TGME49_248760* and *TGME49_257130* were specifically highly expressed at the oocyst stage (Figure S1D). The four genes were stably expressed across multiple *T. gondii* genotypes, with the highest levels in the Pru strain; they exhibited modest fluctuations during the cell cycle, remained stable during bradyzoite differentiation, and two of them showed oocyst-stage-specific high expression.

The four GOIs exhibit highly stage-specific expression, predominantly in oocyst and enteroepithelial stages (EES) throughout the *Toxoplasma* life cycle (Table 2), whereas they are barely expressed in asexual tachyzoites and bradyzoites within intermediate hosts. Specifically, *TGME49_257130* and *TGME49_248760* were highly expressed in unsporulated and sporulating oocysts, with expression downregulated after sporulation completion. All four genes showed gradually increasing expression during the cat intestinal enteroepithelial stages (EES1–EES5), peaking at the EES5 stage. These results suggest that these genes are involved in gametogenesis and subsequent oocyst development and differentiation during the intestinal sexual development of *T. gondii* within the definitive host intestinal epithelium.

## 4. Discussion

In this study, we employed the CRISPR-Cas9 system to generate individual knockout strains for five previously uncharacterized genes in *T. gondii*, including three CCCH-type zinc finger proteins (*TGME49_257130*, *TGME49_500318*, *TGME49_313740*) and two subtilisin-like serine proteases (*TGME49_248760*/*sub7*, *TGME49_235950*/*sub8*). Diagnostic PCR confirmed the complete replacement of each open reading frame, verifying successful gene disruption at the genomic level. Our systematic phenotypic assessments demonstrated that deletion of these genes does not impair tachyzoite growth, replication, egress, acute virulence in mice, or brain cyst formation. These findings suggest that, unlike other characterized members of their respective families that play essential roles in the asexual lytic cycle, the five genes studied here are dispensable for the tachyzoite stage in vitro and in vivo.

This phenotypic neutrality aligns with predictions of both our own and public transcriptomic data. Our WGCNA of single-cell transcriptomic data of cat intestine after *Toxoplasma* infection and ToxoDB-based analyses revealed that these five GOIs exhibit extremely low transcript abundance in tachyzoites. Consistent with this transcriptional profile, none of the knockout strains showed detectable phenotypic differences from the parental wild-type strain in growth, replication, egress, or virulence. This concordance between transcriptomic predictions and experimental observations serves as a positive validation that the knockout strains are phenotypically neutral in the tachyzoite stage, which is an essential step before reliably deploying these mutants in future feline infection experiments to investigate their sexual-stage functions. We consider that the lack of phenotypic differences in tachyzoites does not diminish the value of these mutants; rather, it confirms that these genes are indeed dispensable in this stage, and ensures that any phenotypes observed in subsequent sexual-stage studies can be attributed to stage-specific functions rather than to pleiotropic defects inherited from the asexual growth phase.

Within the zinc finger protein family, different members exhibit remarkable functional diversity. Previous studies have reported that TgZFP1 localizes to the nucleolus and participates in tachyzoite to bradyzoite differentiation [17]; TgZFP2 localizes to the pericentriolar region and plays a key role in coordinating mitosis and daughter budding [18]; and TgZNF2 is a nuclear protein that interacts with the spliceosome and mRNA export complexes to facilitate nuclear export of polyA^+^ mRNA [19]. However, the three CCCH-type zinc finger proteins analyzed in this study are all non-essential during the tachyzoite stage, consistent with bioinformatic data showing negligible expression in tachyzoites.

CCCH-type zinc finger proteins serve as RNA regulators that govern the stability, localization, and translation of their target mRNAs in eukaryotic organisms [31]. In *Plasmodium*, the CCCH-type zinc finger protein ZNF4 is abundantly expressed in mature gametocytes; its loss does not impair asexual blood-stage proliferation or gametocyte maturation but markedly disrupts male gametocyte exflagellation, thereby decreasing parasite transmission efficiency to mosquitoes [32]. In *Toxoplasma*, BFD2 has been identified as a CCCH-type zinc finger protein, and its deletion reduces the protein levels of the differentiation regulator BFD1, impairing complete differentiation under stress conditions [33,34]. These studies indicate that CCCH-type zinc finger proteins are key regulators of parasite stage conversion and gamete development. In this study, *TGME49_257130* exhibited extremely high expression in unsporulated oocysts (1468.69 TPM), as well as high expression levels in sporulating oocysts, sporulated oocysts, and EES, thus warranting focused attention in future studies. In contrast, *TGME49_313740* showed gradually increasing expression during EES; although the expression level was relatively low, its expression specificity (absent in tachyzoites and bradyzoites, detected only in sexual stages) strongly suggests a stage-specific function. The third CCCH protein (*TGME49_500318*) lacks transcriptomic data, but its similar phenotypic profile (no defect in the tachyzoite stage) implies that it may also participate in the sexual stage. We therefore propose that these three CCCH proteins may regulate the translation or stability of stage-specific genes during the sexual phase through analogous RNA-binding mechanisms, thereby participating in gametogenesis or oocyst formation.

The subtilisin-like protease family in *Toxoplasma* also exhibits functional diversity. TgSUB1 knockout leads to delayed invasion, reduced adhesion, and attenuated virulence in mice [20,22]; TgSUB2 is a rhoptry protein maturase with specificity similar to that of ROP1, a virulence factor that diminishes the parasite’s sensitivity to innate immune restriction in both mice and humans [23,35]; TgSUB3 deletion results in significantly impaired plaque formation and reduced tachyzoite numbers per vacuole [25]. In contrast, SUB7 and SUB8 examined in this study showed no phenotype in tachyzoites, consistent with their extremely low transcript levels in tachyzoites, suggesting that their functions may be specifically restricted to sexual reproduction. Oocyst formation and sporulation involve complex processes of cell differentiation, wall formation, and metabolic reprogramming. Multiple studies have revealed that a large number of genes are specifically upregulated during oocyst development. These genes may participate in the processing of oocyst wall proteins, metabolic reprogramming during sporulation, or the establishment of sporozoite infectivity [36,37]. Similar functional specificity has been reported for other genes, such as Grx5 knockout, which shows no phenotype during asexual stages but significantly affects oocyst production and sporulation [38]. Analyses of transcriptomic data from the ToxoDB database revealed upregulation of SUB7 and SUB8 during feline enteric stages, with transcript levels increasing from 1.46 to 496.58 TPM and from 5.95 to 105.36 TPM, respectively, between EES1 and EES5. This progressive surge, particularly for SUB7 at late stages, implies their active roles in gamete fusion, zygote formation, or oocyst maturation. Given that subtilisin proteases function in proteolytic cascades for organelle and wall formation in other apicomplexans [20,21], SUB7 and SUB8 may participate in processing specific substrates in the sexual stage, which need experimental validation.

Our phenotypic analyses indicate that these five GOIs are not essential for basic biological functions in tachyzoites, thereby providing clear boundaries for further functional investigation. High expression of a gene at a specific stage often implies a function at that stage. In this study, four of the five GOIs, with high transcript levels in oocyst or enteroepithelial stages but barely detectable expression in tachyzoite or bradyzoite stages (transcriptional levels obtained from the ToxoDB database), exhibited no detectable phenotype in tachyzoite stages. Importantly, the absence of detectable phenotypes in tachyzoite growth, virulence, and cyst formation does not imply functional insignificance. When interpreted together with the transcriptomic data showing preferential expression in oocyst and feline enteroepithelial stages, these results suggest that the five genes likely participate in the sexual stage of *T. gondii*, potentially through involvement in sexual differentiation, gamete formation, or oocyst maturation, which were not evaluated in our current assays. This stage-specific expression-phenotype concordance provides a clear rationale for directing future investigations toward feline infection models to directly define their roles in these processes.

Recent investigations have yielded significant insights into elucidating the molecular regulation of sexual reproduction in *Toxoplasma*. Studies have shown that the MORC/HDAC3 complex represses the expression of sexual stage genes during the asexual stage through epigenetic silencing [39,40]. AP2XII-2 has been identified as an important transcription factor involved in repressing sexual stage gene expression, regulating stage-specific genes including AP2X-10 [41]. AP2XII-1 and AP2XI-2 operate during the tachyzoite stage to silence genes required for merozoite development [42,43]. Together, these studies reveal that sexual development in *Toxoplasma* is governed by a complex regulatory network. We propose that the expression of the five genes characterized in this study may be regulated by a similar epigenetic silencing mechanism: they are maintained at low levels during the asexual reproduction in the intermediate host, and this silencing is relieved in the feline intestinal environment, thereby allowing gene expression and initiating sexual developmental program. This hypothesis is further supported by analogous cases, such as Grx5 [38] and AP2XI-2 [42], whose asexual-stage disruption yields no phenotype but whose expression and function are confined to sexual stages, underscoring the correlation between transcriptional silencing in intermediate hosts and stage-specific functionality in the definitive host. However, this inference is currently correlative and remains to be experimentally validated, for instance, by examining whether perturbation of the epigenetic silencing machinery, through genetic or chemical approaches [44,45], leads to their derepression in tachyzoites. Such approaches would help distinguish whether their silencing is actively maintained by the epigenetic machinery or is an intrinsic property of the developmental program.

This study is phenotypic in nature, and the phenotyping data presented here may serve as a reference and foundation for future mechanistic investigations, particularly regarding the potential roles of these genes in sexual development of *T. gondii*. Drawing on the results of these findings, future studies could focus on the following aspects. First, cat infection experiments should be conducted to assess the impact of knockout strains on infectivity in cats, oocyst production, oocyst morphology, and sporulation capacity, thereby directly validating their functions during the sexual stage [46]. Second, RNA-seq or proteomics can be used in a cat infection model to analyze changes in gene expression in knockout strains and identify downstream pathways regulated by these genes [39]. Third, for SUB7 and SUB8, yeast two-hybrid or immunoprecipitation techniques can be employed to screen for protein substrates (e.g., oocyst wall proteins) processed during the sexual stage [37,42]; for CCCH proteins, CLIP-seq or RNA immunoprecipitation can be used to identify their RNA targets [33,47].

## 5. Conclusions

In this study, we successfully generated five knockout strains for three CCCH-type zinc finger protein genes and two subtilisin-like protease genes in *T. gondii* type II Pru strain using the CRISPR-Cas9 system. The absence of detectable phenotypic differences between these mutants and the parental strain in tachyzoites, which is consistent with their extremely low transcript abundance at this stage, confirms successful gene disruption without unintended growth defects. These well-characterized knockout strains provide valuable tools for future investigations into the roles of these genes in the sexual stages of *T. gondii*, particularly in feline intestinal epithelial development and oocyst formation.

## Figures and Tables

**Table 1:** Bioinformatic features of five *T. gondii* GOIs.

Gene ID	Product Description	Exons	Phenotype Value	Mol wt (kDa)	Predicted Signal Peptide	TMHMM *
*TGME49_257130*	zinc finger (CCCH type) motif-containing protein	3	0.79	88.803	no	no
*TGME49_500318*	zinc finger (CCCH type) motif-containing protein	4	N/A ^†^	105.475	no	no
*TGME49_313740*	zinc finger (CCCH type) motif-containing protein	3	1.11	136.899	no	no
*TGME49_248760*	subtilisin SUB7	4	−0.43	62.549	no	yes
*TGME49_235950*	subtilisin SUB8	4	0.82	219.076	no	no

* Transmembrane helices were predicted using TMHMM version 2.0. ^†^ N/A, not available (no phenotype value recorded in the ToxoDB database at the time of analysis).

**Table 2:** Full life-cycle transcriptomic characteristics of four *T. gondii* GOIs ^*^.

Gene ID	Unsporulated *	Sporulating *	Sporulated *	Tachyzoites *	Tissue Cysts *	EES1 *	EES2 *	EES3 *	EES4 *	EES5 *
*TGME49_257130*	1468.69	967	146.23	0.41	0.15	2.3	25.61	38.65	86.2	263.44
*TGME49_313740*	3.61	2.22	8.7	0	0	3.82	7.66	11.63	10.89	26.95
*TGME49_248760*	2740.1	2976.54	126.35	0.12	0.4	1.46	30.66	60.82	163.48	496.58
*TGME49_235950*	3.41	8.29	5.43	0.08	0.32	5.95	19.4	28.43	34.65	105.36

* Transcript abundance is quantified as transcripts per million (TPM).

## Data Availability

The original contributions presented in this study are included in the article and Supplementary Materials. Further inquiries can be directed to the corresponding authors. The transcriptomic data re-analyzed in this study were derived from the publicly accessible ToxoDB database (release 68; https://toxodb.org; accessed on 1 March 2026).

## Supplementary Materials

The following supporting information can be downloaded at https://www.mdpi.com/article/10.3390/ani16172704/s1. Table S1: Primers used in the construction of the GOIs knock-out strains. Figure S1: (A) Transcriptional profiles of GOIs across different *T. gondii* genotypes. (B) Transcriptional profiles of GOIs throughout the cell cycle. (C) Transcriptional profiles of GOIs during bradyzoite differentiation. (D) Transcriptional profiles of GOIs at distinct developmental stages.
