# Supplementary material for: Asexual Development and Virulence Are Not Affected by Knockout of Five Sexual-Stage Candidate Genes in Toxoplasma gondii
Source: Animals (Basel). 2026 Aug 31;16(17):2704. doi: 10.3390/ani16172704 (PMC13564330; doi:10.3390/ani16172704)
Supplement: Supplementary file 1 [file animals-16-02704-s001.zip › Table S1.pdf]

Table S1 Primers used in the construction of the GOIs knock-out strains.

Primer	Sequence (5′-3′)	Use
SgRNA-TGME49_257130-KO	TGGAACACCAGAGTTTGCGG	SgRNA of the CRISPR plasmid for deleting TGME49_257130
Sg-TGME49_257130-KO-Fw	TGGAACACCAGAGTTTGCGGGTTTTAGAGCTAGAAATAGC	Construct the CRISPR plasmid for deleting TGME49_257130
SgRNA-Rv	AACTTGACATCCCCATTTAC	Construct the CRISPR plasmid for deleting GOIs
U5-TGME49_257130-Gbison-Fw	GGTTTTCCCAGTCACGACGTTGTTCGGGCGGTACACCATA	Amplify the 5' homologous arms of TGME49_257130
U5-TGME49_257130-Gbison-Rv	GGATTTACAGCCTGGCGAAGCTTGCGGAATCCTGACGGATAT	Amplify the 5' homologous arms of TGME49_257130
U3-TGME49_257130-Gbison-Fw	CTATGCACTTGCAGGATGAATTCGAGAAATCGAGCCGCAAC	Amplify the 3' homologous arms of TGME49_257130
U3-TGME49_257130-Gbison-Rv	GAGCGGATAACAATTTCACATGTTGAAGGAAGGTATGCACA	Amplify the 3' homologous arms of TGME49_257130
DHFR-Gibson-Fw	AAGCTTCGCCAGGCTGTAAATCC	Amplify the DHFR fragment
DHFR-Gibson-Rv	GAATTCATCCTGCAAGTGCATAG	Amplify the DHFR fragment
pUC19-Gibson-Fw	TGTGAAATTGTTATCCGCTC	Amplify the pUC19 fragment
pUC19-Gibson-Rv	AACGTCGTGACTGGGAAAACC	Amplify the pUC19 fragment
5H-TGME49_257130-KZ-Fw	GTTCGGGCGGTACACCATA	Amplify the 5H-DHFR-3H fragment of TGME49_257130
3H-TGME49_257130-KZ-Rv	TGTTGAAGGAAGGTATGCACA	Amplify the 5H-DHFR-3H fragment of TGME49_257130
PCR1-TGME49_257130-Fw	CCCATCTTTCTAGCCGAAGAT	Detect the insertion of 5' homologous fragment of TGME49_257130 in PCR1
PCR1-DHFR-Rv	GCCAAAGTAGAAAGGAATTAGCAT	Detect the insertion of 5' homologous fragment of TGME49_257130 in PCR1
PCR2-TGME49_257130-Fw	GGCGTGTTTGCTTTCCTG	Detect the deletion of TGME49_257130 in PCR2
PCR2-TGME49_257130-Rv	ACTGTATTCGGTGTTGCACC	Detect the deletion of TGME49_257130 in PCR2

PCR3-DHFR-Fw	TGACGCAGATGTGCGTGTATCCAC	Detect the insertion of 3' homologous fragment of TGME49_257130 in PCR3
PCR3-TGME49_257130-Rv	TTTTCGGGCACTAGGAACCA	Detect the insertion of 3' homologous fragment of TGME49_257130 in PCR3
SgRNA-TGME49_500318-KO	CTCAAACAACATCACCAGAG	SgRNA of the CRISPR plasmid for deleting TGME49_500318
Sg-TGME49_500318-KO-Fw	CTCAAACAACATCACCAGAGGTTTTAGAGCTAGAAATAGC	Construct the CRISPR plasmid for deleting TGME49_500318
U5-TGME49_500318-Gbison-Fw	GGTTTTCCCAGTCACGACGTTGACGAGCGAAAAAGGGAGATAG	Amplify the 5' homologous arms of TGME49_500318
U5-TGME49_500318-Gbison-Rv	GGATTTACAGCCTGGCGAAGCTTGTCTTGTAAAATTTGGTCGTCCG	Amplify the 5' homologous arms of TGME49_500318
U3-TGME49_500318-Gbison-Fw	CTATGCACTTGCAGGATGAATTCGGTCTCTTGAGGTTGTCCAAC	Amplify the 3' homologous arms of TGME49_500318
U3-TGME49_500318-Gbison-Rv	GAGCGGATAACAATTTCACACCAGACGATGAGGGAACAG	Amplify the 3' homologous arms of TGME49_500318
5H-TGME49_500318-KZ-Fw	GACGAGCGAAAAAGGGAGATAG	Amplify the 5H-DHFR-3H fragment of TGME49_500318
3H-TGME49_500318-KZ-Rv	CCAGACGATGAGGGAACAG	Amplify the 5H-DHFR-3H fragment of TGME49_500318
PCR1-TGME49_500318-Fw	ACGAGGTCTCTTTTCCGTAC	Detect the insertion of 5' homologous fragment of TGME49_500318 in PCR1
PCR2-TGME49_500318-Fw	ATTCCACGAGAAGAGAACGC	Detect the deletion of TGME49_500318 in PCR2
PCR2-TGME49_500318-Rv	CACGTCAGTCGTTGTGCT	Detect the deletion of TGME49_500318 in PCR2
PCR3-TGME49_500318-Rv	AGAGACTTGGCAGCAAGC	Detect the insertion of 3' homologous fragment of TGME49_500318 in PCR3
SgRNA-TGME49_313740-KO	ATTTGCGACCTTCACCGACA	SgRNA of the CRISPR plasmid for deleting TGME49_313740
Sg-TGME49_313740-KO-Fw	ATTTGCGACCTTCACCGACAGTTTTAGAGCTAGAAATAGC	Construct the CRISPR plasmid for deleting TGME49_313740
U5-TGME49_313740-Gbison-Fw	GGTTTTCCCAGTCACGACGTTAAAGATGACTTTCCAGCAGGA	Amplify the 5' homologous arms of TGME49_313740
U5-TGME49_313740-Gbison-Rv	GGATTTACAGCCTGGCGAAGCTTCTGTTGAAGGATCCCAGAATCAA	Amplify the 5' homologous arms of TGME49_313740
U3-TGME49_313740-Gbison-Fw	CTATGCACTTGCAGGATGAATTCCTTGAAAGGACCTCTCCGT	Amplify the 3' homologous arms of TGME49_313740

U3-TGME49_313740-Gbison-Rv	GAGCGGATAACAATTTCACAGCTTTGATTACGGGTTTGTGC	Amplify the 3' homologous arms of TGME49_313740
5H-TGME49_313740-KZ-Fw	AAAGATGACTTTCCAGCAGGA	Amplify the 5H-DHFR-3H fragment of TGME49_313740
3H-TGME49_313740-KZ-Rv	GCTTTGATTACGGGTTTGTGC	Amplify the 5H-DHFR-3H fragment of TGME49_313740
PCR1-TGME49_313740-Fw	CGTACCTGAGCTTGGGTT	Detect the insertion of 5' homologous fragment of TGME49_313740 in PCR1
PCR2-TGME49_313740-Fw	GTACTGAACAGTGGCGGTG	Detect the deletion of TGME49_313740 in PCR2
PCR2-TGME49_313740-Rv	CAACTTATTTTGGGGGGACCT	Detect the deletion of TGME49_313740 in PCR2
PCR3-TGME49_313740-Rv	TCACGAATCCTGCTCCAAAC	Detect the insertion of 3' homologous fragment of TGME49_313740 in PCR3
SgRNA-TGME49_248760-KO	GGCGTCTGTTGAGTACGATG	SgRNA of the CRISPR plasmid for deleting TGME49_248760
Sg-TGME49_248760-KO-Fw	GGCGTCTGTTGAGTACGATGGTTTTAGAGCTAGAAATAGC	Construct the CRISPR plasmid for deleting TGME49_248760
U5-TGME49_248760-Gbison-Fw	GGTTTTCCCAGTCACGACGTTGTCGAATCTTTCACAGAGCCT	Construct the CRISPR plasmid for deleting TGME49_248760
U5-TGME49_248760-Gbison-Rv	GGATTTACAGCCTGGCGAAGCTTTGTGGCAGCAAATGCGAT	Amplify the 5' homologous arms of TGME49_248760
U3-TGME49_248760-Gbison-Fw	CTATGCACTTGCAGGATGAATTCGCAAGCAGGTCACTTTCTTG	Amplify the 3' homologous arms of TGME49_248760
U3-TGME49_248760-Gbison-Rv	GAGCGGATAACAATTTCACACTGCAACAGCTGCTGAGA	Amplify the 3' homologous arms of TGME49_248760
5H-TGME49_248760-KZ-Fw	GTCGAATCTTTCACAGAGCCT	Amplify the 5H-DHFR-3H fragment of TGME49_248760
3H-TGME49_248760-KZ-Rv	CTGCAACAGCTGCTGAGA	Amplify the 5H-DHFR-3H fragment of TGME49_248760
PCR1-TGME49_248760-Fw	AATCACTTGTTGCTACATCGG	Detect the insertion of 5' homologous fragment of TGME49_248760 in PCR1
PCR2-TGME49_248760-Fw	ACAGACGCGCCAAATGAT	Detect the deletion of TGME49_248760 in PCR2
PCR2-TGME49_248760-Rv	AAGATACCCCCGTGTCGAT	Detect the deletion of TGME49_248760 in PCR2
PCR3-TGME49_248760-Rv	GGCGTGTATCTACTCGTGAGA	Detect the insertion of 3' homologous fragment of TGME49_248760 in PCR3

SgRNA-TGME49_235950-KO	CGGTGTATTCAGAACACACG	SgRNA of the CRISPR plasmid for deleting TGME49_235950
Sg-TGME49_235950-KO-Fw	CGGTGTATTCAGAACACACGGTTTTAGAGCTAGAAATAGC	Construct the CRISPR plasmid for deleting TGME49_235950
U5-TGME49_235950-Gbison-Fw	GGTTTTCCCAGTCACGACGTTTTCTGGTGTAGAACGTCGGTT	Amplify the 5' homologous arms of TGME49_235950
U5-TGME49_235950-Gbison-Rv	GGATTTACAGCCTGGCGAAGCTTCGCAACCTGTAGTACTGAGAGA	Amplify the 5' homologous arms of TGME49_235950
U3-TGME49_235950-Gbison-Fw	CTATGCACTTGCAGGATGAATTCCTTCCACCTGGTGTGTACG	Amplify the 3' homologous arms of TGME49_235950
U3-TGME49_235950-Gbison-Rv	GAGCGGATAACAATTTCACACAGGAGGACAAGCAAGCAG	Amplify the 3' homologous arms of TGME49_235950
5H-TGME49_235950-KZ-Fw	TTCTGGTGTAGAACGTCGGTT	Amplify the 5H-DHFR-3H fragment of TGME49_235950
3H-TGME49_235950-KZ-Rv	CAGGAGGACAAGCAAGCAG	Amplify the 5H-DHFR-3H fragment of TGME49_235950
PCR1-TGME49_235950-Fw	AAACACTCACAGCAGGTCATT	Detect the insertion of 5' homologous fragment of TGME49_235950 in PCR1
PCR2-TGME49_235950-Fw	TGTTGAAAAGCTATGCCAGC	Detect the deletion of TGME49_235950 in PCR2
PCR2-TGME49_235950-Rv	CCGGAGCGAGAAAGAGAA	Detect the deletion of TGME49_235950 in PCR2
PCR3-TGME49_235950-Rv	GGCAATGTGTTTCTGTGGG	Detect the insertion of 3' homologous fragment of TGME49_235950 in PCR3
